# Clinical Characteristics and Outcomes of Endemic Mycoses After Solid Organ Transplantation: A Comprehensive Review

**DOI:** 10.1093/ofid/ofae036

**Published:** 2024-01-22

**Authors:** Cybele Lara R Abad, Raymund R Razonable

**Affiliations:** Department of Medicine, Section of Infectious Diseases, University of the Philippines Manila, Philippine General Hospital, Manila, Philippines; Department of Medicine, Division of Public Health, Infectious Diseases and Occupational Medicine, and The William J Von Liebig Center for Transplantation and Clinical Regeneration, Mayo Clinic College of Medicine and Sciences, Rochester, Minnesota, USA

**Keywords:** *Blastomyces*, *Coccidioides/paracoccidioides*, *Histoplasma*, *Talaromyces*, transplantation

## Abstract

**Background:**

Geographically endemic fungi can cause significant disease among solid organ transplant (SOT) recipients. We provide an update on the epidemiology, clinical presentation, and outcomes of 5 endemic mycoses in SOT recipients.

**Methods:**

Multiple databases were reviewed from inception through May 2023 using key words for endemic fungi (eg, coccidioidomycosis or *Coccidioides,* histoplasmosis or *Histoplasma,* etc). We included adult SOT recipients and publications in English or with English translation.

**Results:**

Among 16 cohort studies that reported on blastomycosis (n = 3), coccidioidomycosis (n = 5), histoplasmosis (n = 4), and various endemic mycoses (n = 4), the incidence rates varied, as follows: coccidioidomycosis, 1.2%–5.8%; blastomycosis, 0.14%–0.99%; and histoplasmosis, 0.4%–1.1%. There were 204 reports describing 268 unique cases of endemic mycoses, including 172 histoplasmosis, 31 blastomycosis, 34 coccidioidomycosis, 6 paracoccidioidomycosis, and 25 talaromycosis cases. The majority of patients were male (176 of 261 [67.4%]). Transplanted allografts were mostly kidney (192 of 268 [71.6%]), followed by liver (n = 39 [14.6%]), heart (n = 18 [6.7%]), lung (n = 13 [4.9%]), and combined kidney-liver and kidney-pancreas (n = 6 [2.7%]). In all 5 endemic mycoses, most patients presented with fever (162 of 232 [69.8%]) and disseminated disease (179 of 268 [66.8%]). Cytopenias were frequently reported for histoplasmosis (71 of 91 [78.0%]), coccidioidomycosis (8 of 11 [72.7%]) and talaromycosis (7 of 8 [87.5%]). Graft loss was reported in 12 of 136 patients (8.8%). Death from all-causes was reported in 71 of 267 (26.6%); half of the deaths (n = 34 [50%]) were related to the underlying mycoses.

**Conclusions:**

Endemic mycoses commonly present with fever, cytopenias and disseminated disease in SOT recipients. There is a relatively high all-cause mortality rate, including many deaths that were attributed to endemic mycoses.

## Teaser

Endemic mycoses may cause severe disease among SOT recipients, especially those with epidemiologic risk. Fever, disseminated disease, and cytopenia are common. Heightened suspicion for these mycoses is needed to facilitate early recognition and reduce morbidity and mortality rates in SOT recipients.

## Graphical Abstract

**Graphical Abstract:**
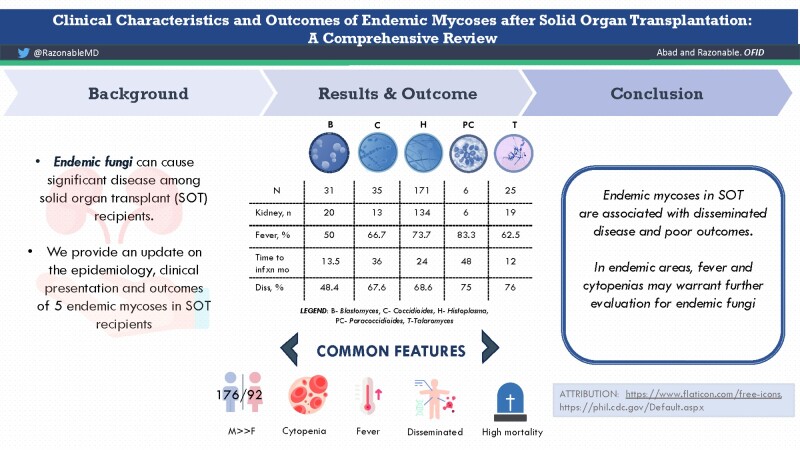

*Blastomyces* spp, *Coccidioides* spp, *Histoplasma* spp, *Paracoccidioides* spp, and *Talaromyces* spp (formerly *Penicillium* spp*)* are geographically restricted fungi collectively known as *endemic mycoses*. Solid organ transplant (SOT) recipients living in affected regions may be at risk of infection. With widespread travel, SOT donors and recipients in nonendemic regions may be exposed to these fungi leading to sporadic cases globally.

Published literature on endemic mycoses in SOT are limited to case reports or series from single institutions. Although there have been a few comprehensive reviews [1–4], these are usually limited to just a single fungus. We aimed this encompassing review to (1) provide an update on the epidemiology, clinical characteristics, and outcomes of different endemic fungi after transplantation and (2) highlight common attributes and identify potentially unique characteristics that may be useful to transplant clinicians unfamiliar with these geographically restricted fungal infections.

## METHODS

### Literature Search

Using multiple databases—EBM Reviews, —Cochrane Central Register of Controlled Trials, EBM Reviews—Cochrane Database of Systematic Reviews, Embase, Ovid MEDLINE and Epub Ahead of Print, In-Process, In-Data-Review & Other Non-Indexed Citations, Daily and Versions, SCOPUS, and Web of Science from inception through May 2023—we identified all cases of endemic fungi after transplantation. We searched titles and abstracts using key words blastomycos* or *Coccidioides* or “*Coccidioides immitis*” or “*Coccidioides posadasii*” or coccidioidomycosis or coccidiomycos* or “endemic fungi” or “endemic mycos*” or histoplasmos* or paracoccidiomycos* or “*T marneffei*” or *Talaromyces* or “*Talaromyces marneffei*” or “*Penicilium marneffi*” or “penicilliosis.”

Article references were extensively reviewed for additional cases. Our review was limited to adults, SOT, and publications in English or English translation. We included all case reports, case series, and cohorts for as long as the diagnosis of an endemic fungal infection was established in the SOT population. Eligible reports were screened by both authors (C. L. R. A. and R. R. R.). We excluded abstracts, pediatric cases, hematopoietic transplants, and cases of endemic fungi diagnosed before transplantation. Duplicates, reports with mixed non-SOT populations, those with other fungal infections (eg, *Candida* spp, *Cryptococcus* spp, *Aspergillus* spp), and where patient-level information could not be extracted were likewise excluded. Data was coded in an Excel spreadsheet (C. L. R. A.).

A patient was considered to have an endemic fungal infection if at least one of these requirements was met: a positive culture, positive serum or urine antigen (eg, for *Histoplasma*, *Blastomyces*), histopathology demonstrating structures characteristic of the endemic fungi (eg, spherules for *Coccidioides* spp, broad-based budding for *Blastomyces* spp), the presence of H or M precipitin bands by immunodiffusion or complement fixation for *Histoplasma capsulatum* or *Coccidioides* spp, a positive nucleic acid amplification test or DNA probe test result, or metagenomic or next-generation sequencing.

Disseminated infection was defined as clinical, laboratory, or imaging evidence of extrapulmonary involvement or involvement of noncontiguous sites. Pulmonary infection was defined as respiratory symptoms and chest imaging (radiography or computed tomography) with infiltrates and/or mediastinal lymphadenopathy in the absence of infection elsewhere. Extrapulmonary involvement (eg, cutaneous/skin and soft tissue or gastrointestinal tract) was determined based on clinical signs or laboratory evidence.

This study did not include factors necessitating patient consent.

### Statistical Analysis

Detailed case reports and cohorts are described separately, according to the type of endemic fungus. We used summary statistics (eg, median, range, and percentage) to describe categorical and continuous variables. The incidence of the endemic mycoses per study, where applicable, was calculated using the following formula: total number of cases/total number of transplanted population ×100.

## RESULTS

**Figure 1.**
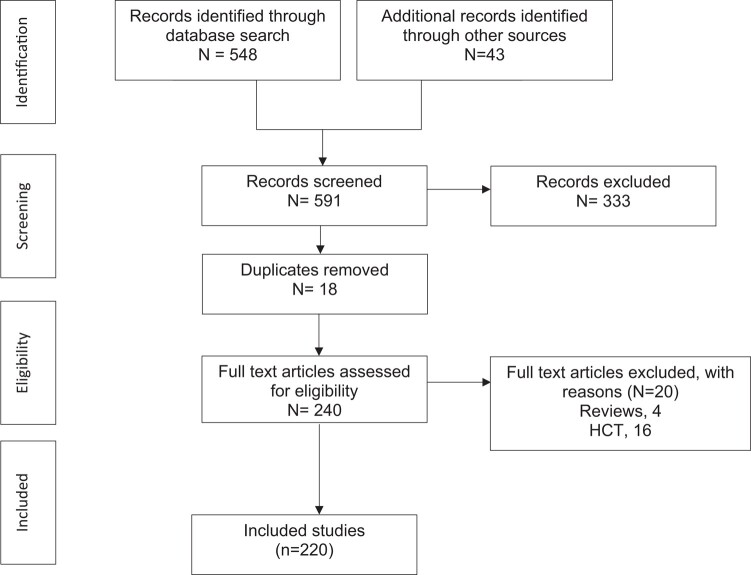
Study flow chart. Abbreviation: HCT, hematopoietic transplant

We screened 548 studies, of which 197 fulfilled criteria [2–197]. Of these, 4 included a review of related literature [2–4, 198], 15 were cohort studies [10, 32, 47, 53, 54, 56, 57, 62, 63, 71, 89, 100, 184, 185, 187], and the majority were case reports. After careful review of references, 1 cohort [1] and 42 additional case reports or series were identified [199–213] including 20 [188, 190, 192, 214–230] from a prior review [4]. (Supplementary Material, Appendix 1; Figure 1).

### Case Reports

A total of 268 SOT recipients with an endemic mycoses were described in 204 reports [4–8, 13, 16, 18, 20, 21, 23, 25, 27, 28, 33–37, 40, 42, 45, 46, 48–52, 55, 58–61, 64–70, 72–88, 90–99, 101–146, 148–168, 170–183, 186, 188–197, 207, 209–244]. Table 1 summarizes their characteristics.

**Table 1.** Baseline Characteristics of Solid Organ Transplant Recipients With Endemic Fungal Infection

	Patients by Infection Type, No. (%)^a^					
Characteristic	Total	Histoplasmosis	Blastomycosis	Coccidioidomycosis	Paracoccidioidomycosis	Talaromycosis
Age, median (range), y	49 (18–81)	47 (18–81)	55 (22–78)	48 (18–70)	51(29–66)	48.5 (33–67)
Male sex	176 (67.4)	110 (65.5)	22 (78.6)	20 (58.8)	4 (66.7)	20 (80)
Studies by region	204 (100)	125 (100)	24 (100)	25 (100)	6 (100)	24 (100)
Africa	1 (0.5)	0	1 (4.2)	0	0	0
Asia/Pacific	48 (23.5)	25 (20)	1 (4.2)	0	0	22 (91.7)
Europe	13 (6.4)	6 (4.8)	2 (8.3)	4 (16)	0	1 (4.2)
North America (USA, Canada, Mexico)	119 (58.3)	76 (60.8)	20 (83.3)	21 (84)	1 (16.7)	1 (4.2)
South America	23 (11.3)	18 (14.4)	0	0	5 (83.3)	0
Transplant type	268 (100)	172 (100)	31 (100)	34 (100)	6 (100)	25 (100)
Kidney	192 (71.6)	134 (77.9)	20 (64.5)	13 (38.2)	6 (100)	19 (76)
Liver	39 (14.6)	23 (13.4)	3 (9.7)	10 (29.4)	0	3 (12)
Heart	18 (6.7)	10 (5.8)	6(19.4)	2 (5.9)	0	0
Lung	13 (4.9)	2 (1.2)	2 (6.4)	6 (17.6)	0	3 (12)
Other organ(s)	6 (2.2)	3 (1.7)	0	3 (8.8)	0	0
Maintenance immune suppression, SOT	230 (100)	144 (100)	29 (100)	29 (100)	6 (100)	22 (100)
MMF	131 (57)	74 (51.4)	19 (65.5)	19 (65.5)	2 (33.3)	17 (77.3)
Non-MMF regimen	99 (43)	70 (48.6)	10 (34.5)	10 (34.5)	4 (66.7)	5 (22.7)
T-cell–depleting agent for induction	72 (100)	51 (100)	7 (100)	13 (100)	1 (100)	NR
Yes	34 (47.2)	23 (45.1)	5 (71.4)	6 (46.2)	1 (16.7)	0
No	38 (52.8)	28 (54.9)	2 (28.6)	7 (53.8)	5 (83.3)	0
Risk factors	131 (100)	68 (100)	15 (100)	17 (100)	6 (100)	25 (100)
Travel/residence in endemic area	100 (76.3)	49 (72.1)	7 (46.7)	15 (88.2)	4 (66.7)	25 (100)
Work related only	4 (3.1)	3 (4.4)	1 (6.7)	0	0	0
Both	27 (20.6)	16 (23.5)	7 (46.7)	2 (11.8)	2 (33.3)	0
Symptom onset since SOT	239 (100)	155^b^ (100)	26 (100)	29 (100)	6 (100)	23 (100)
Time to symptom onset, median (range), mo	24 (0.2–360)	24 (0.4–360)	13.5(0.43–168)	36 (0.2–108)^c^	48 (18–168)	12(0.5–140)
Early (<12 mo)	106 (46.2)	59 (40.4)	13 (50)	20 (68.9)	1 (16.7)	13 (56.5)
Late (≥12 mo)	124 (53.8)	87 (59.6)	13 (50)	9 (31.1)	5 (83.3)	10 (43.5)
Acute rejection	45/91 (49.4)	30/65 (46.2)	6/7 (85.7)	6/14 (42.9)	0/1 (0)	3/4(75)
Type of infection	268 (100)	172 (100)	31 (100)	34 (100)	6 (100)	25 (100)
Pulmonary	58 (21.6)	31(18)	12 (38.7)	10 (29.4)	2 (25)	3 (12)
Disseminated	179 (66.8)	118 (68.6)	15 (48.4)	23 (67.6)	4 (75)	19 (76)
Cutaneous	21 (7.8)	14 (8.1)	4 (12.9)	0	0	3 (12)
Other	9 (3.4)	9 (5.3)^d^	0	0	0	0
Asymptomatic	1 (0.4)	0	0	1 (3)	0	0
Fever	162/232 (69.8)	115/156 (73.7)	11/22 (50)	16/24 (66.7)	5/6 (83.3)	15/24 (62.5)
Diagnosis method	261 (100)	168 (100)	31 (100)	34 (100)	6 (100)	22 (100)
Culture alone	52 (19.9)	30 (17.9)	8(25.8)	7 (20.6)	0	7 (31.8)
Histopathology alone	83 (31.8)	62 (36.9)	7 (22.6)	5 (14.7)	3 (50)	6 (27.3)
Serology alone	16 (6.1)	7 (4.2)	0	9 (26.5)	0	0
Combination (any)	110 (42.1)	69 (41.1)	16(51.6)	13 (38.2)	3 (50)	9 (40.9)
Additional PCR, DNA probe, or NGS as diagnostic	17	8	2	2	0	5
Chest imaging	183/259 (70.7)	127/164 (77.4)	24/31 (77.4)	20/34 (58.8)	6/6 (100)	6/24 (25)
Abnormal findings	139 (76.0)	91 (71.7)	23 (95.8)	15 (75)	5 (83.3)	5 (83.3)
Normal	44 (24.0)	36 (28.3)	1 (4.2)	5 (25)	1 (16.7)	1 (16.7)
Initial treatment	236 (100)	151(100)	30 (100)	29 (100)	5 (100)	21 (100)
AMB preparation (any)	158 (66.9)	108 (71.5)	25 (83.3)	10 (34.6)	4 (80)	11 (52.4)
Azole	71 (30.0)	40 (26.5)	5 (16.7)	15 (51.7)	1 (20)	10 (47.6)
Both	6 (2.5)	3 (2.0)	0	3 (10.3)	0	0
Echinocandin	1 (0.4)	0	0	1 (3.4)	0	0
None/NR	32	21	1	5	1	4
Overall treatment duration, median (range), mo	12 (0.03 to Ind)	12 (0.47 to Ind)	12 (1 to Ind)	0.7 (0.03 to Ind)	3 (0.2–12)	6 (0.47 to Ind)
Reduction in IS	76/95 (80.0)	52/59 (88.1)	5/6 (83.3)	7/7 (100)	NR	12/23 (52.2)
Graft dysfunction/loss	12/136 (8.8)	10/111 (9)	2/25 (8)	0	0	0
Death	71/267 (26.6)	42/171 (24.6)	7/31 (22.6)	11/34 (32.3)	3/6 (50)	8/25 (32)
Related	36 (50.7)	20 (47.6)	6 (85.7)	8 (72.7)	2 (66.7)	0
Unrelated	23 (32.4)	20 (47.6)	1 (14.3)	0	1 (33.3)	1 (12.5)
NR	12 (16.9)	2 (4.8)	0	3 (28.3)	0	7 (87.5)

Abbreviations: AMB, amphotericin B; Ind, indefinite; IS, immune suppression; MMF, mycophenolate mofetil; NGS, next-generation sequencing; NR, not reported; PCR, polymerase chain reaction; SOT, solid organ transplant.

^a^Data represent no. (%) of SOT recipients unless otherwise specified.

^b^Nine not specified (range, 8–120).

^c^Excluding donor-derived infection.

^d^Gastrointestinal (n = 8) or lymph node (n = 1).

### Blastomycosis

Thirty-one cases of blastomycosis were reported [7, 8, 13, 20, 21, 23, 35, 45, 46, 48, 51, 52, 55, 59, 60, 64, 65, 67, 68, 70, 195, 209, 211, 244]. The majority of reports from the United States (10 of 16) were within endemic areas [20, 21, 35, 45, 46, 51, 60, 67, 195, 211]. Eleven [8, 13, 23, 35, 48, 52, 64, 65, 68, 70, 209] had documented risk factors for disseminated infection, while this was unknown in 5 cases [7, 55, 59, 211, 244]. Five mentioned receipt of antithymocyte globulin [23, 64, 65, 209, 244], while 6 reported treatment for acute rejection before disease onset [59, 64, 65, 68, 209, 244]. One patient was treated with methylprednisone for worsening graft function [70], and another had chronic cellular rejection [51]. The majority were receiving mycophenolate mofetil (MMF) (19 of 29 [65.5%]) [7, 8, 13, 21, 35, 45, 46, 48, 55, 59, 64, 65, 67, 70, 244].

Among 26 patients with reported disease onset, the median time after transplantation was 13.5 months (range, 0.43–168 months). Fever occurred in half of patients (11 of 22 [50%]) [7, 8, 20, 21, 23, 35, 51, 64, 65, 195, 209]. Slightly less than half had disseminated infection (15 of 31 [48.4%]) [8, 13, 23, 35, 45, 51, 52, 60, 64, 67, 68, 211], followed by pulmonary infection alone (12 of 31 [38.7%]) [7, 20, 21, 46, 67, 70, 195, 209, 211, 244] and isolated cutaneous involvement (4 of 31 [12.9%]) [48, 55, 59, 65]. Sites of dissemination included skin and soft tissue (n = 8), joint (n = 2), central nervous system [CNS] (n = 5), larynx, (n = 2), liver (n = 1), spleen (n = 1), and peritoneum (n = 1). None were fungemic. Chest imaging was performed for 24 of 31 recipients (77.4%) [7, 8, 13, 20, 21, 23, 35, 45, 46, 51, 52, 64, 65, 67, 68, 70, 195, 209, 244] with variable findings, including consolidation (n = 6), diffuse (n = 4) or focal infiltrate or mass (n = 4), nodules (n = 5), cavitation (n = 2), or a combination (n = 2).

For half of cases (16 of 31 [51.6%]), the diagnosis was established using both histopathology and culture (Table 1). *Blastomyces* antibodies was detected in 3 cases [48, 65, 209], and antigen in 1 [8]. In 2 reports [7, 64], DNA probe and cell-free microbial DNA in plasma were used to confirm the diagnosis.

Amphotericin B product was the initial treatment for most patients (25 of 30 [83.3%]) before transition to an azole [7, 8, 13, 35, 45, 46, 48, 51, 52, 55, 59, 60, 64, 67, 68, 70, 195, 209, 211]. The other 5 patients were initially treated with an azole [20, 21, 23, 65, 211], including 3 with pulmonary disease, 1 with isolated cutaneous infection, and 1 with disseminated disease. The duration of treatment for those who survived was frequently ≥12 months [7, 13, 35, 45, 60, 211] or intended as lifelong [23, 60].

Few patients had coinfection [8, 21, 45, 55, 70] (Supplementary Material, Appendix 2). Two patients lost their allografts—1 because of chronic graft rejection [51] and 1 because of disseminated infection [64]. Seven patients died (7 of 31 [22.6%]) [8, 46, 64, 67, 70, 211, 244]; 3 had pulmonary involvement, and 4 had disseminated disease.

### Coccidioidomycosis

Since a comprehensive review [2] in 2001 and a review on kidney-only transplants [198], our search identified 25 additional reports [5, 6, 16, 18, 25, 27, 33, 34, 36, 37, 40, 42, 49, 58, 61, 231–238, 245, 246] describing 34 cases of coccidioidomycosis after SOT (Table 1).

Of 22 recipients with reported race, 10 were white, 6 were African American, 5 were Hispanic, and 1 was Asian [28]. Half of the patients lived in an endemic area (n = 17) [5, 6, 16, 18, 25, 27, 33, 34, 36, 49, 50, 58], while 2 [36, 58] had additional work-related exposures.

Six recipients [27, 36, 232, 237] had documented use of a T-cell–depleting agent for induction immunosuppression. Six reports [5, 27, 49, 234, 236, 238] mentioned treatment for acute rejection before disease presentation. One reported history of chronic rejection [232]. The majority of recipients (19 of 29 [65.5%]) were receiving MMF [6, 25, 27, 33, 34, 36, 42, 58, 231, 232, 236, 237, 247].

Most patients presented within the first year after transplantation (20 of 29 [68.9%]), and most of these early infections were potential donor-derived infection (DDI; 16 of 20 [80%]) [34, 40, 42, 61, 232–238]. The overall median time to infection was 3 months (range, 0.2–108 months). After excluding DDI cases (n = 16), the median time to infection among naturally acquired *Coccidioides* spp was 36 months (range, 0.2–108 months). The majority of recipients (66.7%) presented initially with fever [5, 33, 36, 40, 50, 58, 61, 232, 234–237]. One asymptomatic recipient was identified through surveillance because of a suspected DDI [237]. The majority had disseminated disease (23 of 34 [67.6%]) [5, 6, 16, 27, 28, 34, 36, 37, 40, 58, 61, 231–234, 236, 237, 247]. Sites of dissemination included the CNS (n = 4), skin (n = 4), thyroid (n = 1), and other visceral organs, such as kidney (n = 1), heart (n = 1), and liver or peritoneum (n = 3).

Of recipients with disseminated disease, 12 of 19 (63.2%) were on MMF [6, 34, 36, 58, 231, 232, 236, 237, 247], and one-third (7 of 23 [30.4%]) had fungemia [232, 234, 237]. Eight of 11 [6, 34, 58, 232, 237] with disseminated coccidioidomycosis had anemia (n = 6; median hemoglobin level, 10.4 g/dL), lymphopenia (n = 2; absolute lymphocyte count, 0–470), or thrombocytopenia (n = 2; median platelet count, 76). Chest imaging was performed in 20 of 34 recipients (58.8%) [5, 6, 18, 33, 40, 42, 49, 50, 58, 61, 231–233, 235, 237, 246] with the majority (15 of 20 [75%]) showing abnormal findings, including nodules (n = 5), diffuse infiltrates (n = 2), a combination of these (n = 4), and other findings (n = 4)].

The diagnosis of coccidioidomycosis was confirmed through a combination of histopathology and culture (13 of 34 [38.2%]) [6, 18, 37, 58, 61, 232–236], histopathology alone (5 of 34 [14.7%]) [25, 34, 36, 42, 231], culture alone (7 of 34 [20.6%]) [5, 16, 28, 232, 237, 238], or antibody serology alone (9 of 34 [26.5%]) [27, 33, 49, 50, 237], using combinations of complement fixation (n = 5), immune disk diffusion (n = 1), enzyme immunoassay (n = 3), or unspecified serology (n = 5).

Initial therapy was an azole (n = 15) [5, 6, 27, 33, 40, 49, 61, 234, 235, 237, 238], an amphotericin B product (n = 10) [16, 18, 25, 27, 28, 42, 50, 58, 234, 236], or a combination of both (n = 3) [36, 231, 232]. For those patients who survived, a few were treated for <3 months [36, 42], but the vast majority were either still receiving treatment at the time of the report [27, 231, 237] or were already committed to indefinite therapy [28, 49, 50, 61, 232, 235]. Two-thirds were alive and well at last follow-up (23 of 34 [67.6%]). All deaths occurred in SOT recipients with disseminated coccidioidomycosis [6, 25, 34, 58, 232–234, 236, 237].

### Histoplasmosis

There were 172 cases of histoplasmosis described in 125 reports [66, 69, 72–87, 90–99, 101–182, 194, 196, 197, 199–206, 208, 241–243] (Table 1). Half of recipients (74 of 144 [51.4%]) were on MMF [73, 74, 80, 83–86, 92–94, 96, 98, 101–104, 107–109, 113–116, 118–120, 122, 126, 130, 132–135, 140, 143, 145, 148–150, 152, 153, 155, 156, 159, 161–165, 172–174, 177, 179, 181, 194, 196, 197, 204]. T-cell–depleting induction was reported in 23 cases [78, 104, 107, 108, 110, 113–115, 118, 125, 133, 146, 154, 163] while acute rejection occurred in 30 recipients within 1–3 months before symptom onset [72, 73, 76, 78, 90, 103, 108, 114, 115, 118, 124, 128, 129, 135, 141, 146, 160, 166, 174, 202, 241].

The median time to clinical presentation was 24 months (range, 0.4–360 months) after transplantation. About three-fourths presented initially with fever (115 of 156 [73.7%]). The majority had a diagnosis of disseminated disease (118 of 168 [68.6%]), of whom 37 (30.3%) were fungemic. The most common site of dissemination was skin and soft tissue (n = 26); other sites included bone marrow, liver, kidney, CNS, and the gastrointestinal tract. Cytopenias were documented in 71 of 91 patients (78.0%). Seven had hemophagocytic lymphohistiocytosis (HLH) [72, 98, 103, 108, 123, 176].

Chest radiographs were normal in 36 of 127 recipients. Abnormal findings included diffuse (n = 38) or focal (n = 6) infiltrates, nodular opacities (n = 17), lymphadenopathy (n = 11), or a combination of findings (n = 8). In 10 cases [87, 98, 103, 116, 118, 124, 199, 241], the imaging findings evolved from normal to abnormal during the course of illness.

The diagnosis of histoplasmosis was confirmed through a combination of histopathology and culture (n = 69), histopathology alone (n = 62), culture alone (n = 30), or serology alone (n = 7). *Histoplasma* serology was performed in 73 cases, mostly with antigen (42 of 73 [57.5%]) and, less commonly, antibody testing (24 of 73 [32.9%]) or a combination of antigen and antibody tests (7 of 73 [9.6%]). Urine *Histoplasma* antigen was positive in 35 of 42 cases (83.3%), while serum antigen was positive in 8 of 12 (66.7%).

Amphotericin B product was the most common initial treatment (108 of 151 recipients [71.5%]), followed by azole alone (40 of 151 [26.5%]). Of recipients with disseminated disease and data on treatment (n = 108), amphotericin B product was given to 83 of 108 (76.9%), and azole to 25 of 108 (23.1%). The median duration of treatment for survivors was 1 year (range, 0.47 months to lifelong therapy). One patient treated for 2.6 months had recurrence of tenosynovitis, which required reinitiation of therapy for 11 months [129]. Nineteen coinfections were described (Supplementary Material, Appendix 2) [97, 101, 108, 122, 128, 129, 153, 159, 163, 171, 180, 199, 208, 243]. Of the 42 patients who died [74, 77–79, 81, 82, 84, 87, 90, 95, 108, 115, 127, 128, 139, 141, 142, 148, 153, 155, 159–161, 163, 164, 171, 178, 179, 181, 199–201, 208, 241–243], the majority had disseminated histoplasmosis (39 of 42 [92.9%]).

### Paracoccidioidomycosis

*Paracoccidioides* spp infections were reported in 6 SOT recipients with epidemiologic exposures [183, 186, 207, 210, 212, 213]. The median time to infection was 48 months after SOT (range, 18–168 months). Four recipients had disseminated disease [183, 207, 210, 212] with dissemination to the skin or lymph nodes, while the rest had isolated pulmonary infections [186, 213]. The majority presented with fever, respiratory symptoms and had abnormal chest imaging results (Table 1). None had fungemia.

Diagnoses were established using culture [183, 186, 210] or histopathology [207, 212, 213]; in 1 patient, diagnosis was established by histopathologic finding of the characteristic pilot wheel [207]. Antibody testing supported the diagnosis in 3 patients [186, 207, 210].

Four recipients were initially treated with an amphotericin B product [186, 207, 210, 213], and 1 with azole therapy [183]. The durations of therapy in the 3 patients who had good outcome were 3 and 12 months in 2 patients [186, 213] and unknown in the third [210]. Three patients with disseminated disease died, with 2 deaths directly attributable to paracoccidioidomycosis.

### Talaromycosis (Penicilliosis)

Twenty-five cases of talaromycoses were reported among SOT recipients [4, 188–193, 214–230]. All patients lived in highly endemic areas, except for 2 who travelled to endemic areas before transplantation [188, 190] (Table 1).

The median time to presentation was 12 months after SOT (range, 0.5–140 months). The majority of transplant recipients were on MMF (17 of 22 [77.3%]) [4, 189–191, 215–218, 222–225, 227, 229, 230]. A history of acute (2) [190, 215] or chronic (1) rejection [189] was seen in few patients. The majority presented with fever (15 of 24 [62.5%]) [4, 189, 191–193, 214–218, 222, 225, 227, 229] and disseminated disease (19 of 25 [76.0%]) [4, 188–190, 214–220, 222, 223, 225–229]. Many patients with disseminated disease (13 of 19 [68.4%]) had fungemia [189, 190, 214, 215, 218–220, 222, 223, 225, 226, 229]. Sites of dissemination included the gastrointestinal tract (n = 4), skin (n = 3), peritoneum (n = 1), bone (n = 1), lymph node (n = 2), and urinary tract (n = 5). Seven patients (7 of 8) had cytopenia [4, 190, 191, 215, 218, 220, 225], either with anemia (n = 4), leukopenia (n = 6), or thrombocytopenia (n = 3).

Chest imaging was reported in 6 recipients [4, 191, 215, 218, 220, 225]; all except 1 [218] showed abnormalities including a solid lesion with a focal infiltrate (n = 1), multiple lymphadenopathies (n = 1) nodules (n = 1), or interstitial or bilateral infiltrates (n = 2). More than half reported coinfection (13 of 24 [54.2%]) [4, 190, 192, 216, 218–220, 222–225, 230] (Supplementary Material, Appendix 2).

Diagnosis was made through a combination of culture and histopathology (n = 9) [191, 214, 215, 218, 220–223, 225], culture alone (n = 7) [189, 190, 216, 217, 219, 226, 229], or histopathology alone (n = 6) [188, 192, 193, 224, 227, 230]. Fives studies [191, 214, 218, 223, 228] reported use of a DNA probe (n = 1), next-generation sequencing (n = 3), or metagenomic sequencing (n = 1) to confirm the diagnosis.

Initial therapy was an amphotericin B product (n = 11) [188, 190, 215, 216, 218, 219, 221–224, 226] or an azole (n = 10) [4, 189, 191, 217, 225, 227–230]. The median duration of treatment was 6 months (range, 0.47 month to indefinite therapy) among survivors. The majority of patients (7 of 8) who died had disseminated disease [4, 192, 214, 219, 220, 223, 227].

### Cohort Studies

Among 16 cohort studies, 3 reported on blastomycosis [56, 62, 63], 5 on coccidioidomycosis [10, 32, 47, 53, 71], and 4 on histoplasmosis [1, 54, 89, 248], and 4 evaluated a mixture of endemic mycoses [57, 184, 185, 187]. The majority of cohorts were single-institution studies, except for 4 [47, 57, 184, 185] that involved multiple institutions and included surveillance data [184], or registry data [185]. Two were prospective cohorts [184, 185]. Many included all SOT allografts, but a few reported kidney-only [10, 89, 185] or liver-only [71] recipients. Studies were performed in institutions within endemic areas of the United States and Colombia.

In the cohort studies evaluating blastomycosis, more male than female SOT recipients were affected. The median time to infection was >12 months. Incidence rates for blastomycosis ranged from 0.136 to 0.988. The overall mortality rate ranged between 10.5% and 36.4%.

All coccidioidomycosis cohorts were single-center retrospective studies, except for one [47]. The incidence of infection ranged from 1.2% to 5.8%. The largest study involved 91 patients from 2 institutions in Arizona. For this cohort, all patients received antifungal prophylaxis for 6–12 months. The median time to presentation for this study was 59 months after SOT (range, 25–100 months). The overall mortality rate was 19.78%, but only 1 of 91 recipients died of the underlying infection [47].

Three of the 4 studies on histoplasmosis were relatively small, from single institutions and describing cohorts of 10–23 patients [54, 89, 248]. The largest study [1] included 152 SOT recipients from 24 centers around the United States, including nonendemic areas. In this cohort, the median time to presentation was 27 months (range, 1–240 months). The overall mortality rate was 19.1%, with about half of the rate (9.9%) attributable to histoplasmosis. Only 2 of 4 studies [54, 89] provided data on the incidence of histoplasmosis, which ranged from 0.39% to 1.1%.

The 4 remaining studies looked at a mixture of different infections [57, 184, 185, 187]. A summary of these studies is in Table 2.

**Table 2.** Study Cohorts of Endemic Mycoses

Authors and Year (Country)	Study Period	Allograft	Age, Median (Range), y	Male Sex, No. (Race, No.)	Incidence^a^	Time to Symptoms, Median (Range), mo	Type of Infection	Treatment, No.	Mortality Rate (Attributable), %
Blastomycosis									
Gauthier et al [56] 2007 (USA)	1986–2004	7 K, 3 L, 1 Lu	52 (33–39)	6 (NR)	0.136	26 (0.4–250)	9 P, 4 D, 3 Cut	AMB, 7 > azole,^d^ 4; azole, 4	36.4 (9.1)
McBride et al [62] 2021 (USA)	2004–2016	9 K, 3 L, 3 Lu, 2 KP, 2 H	52 (29–68)	14 (NR)	0.271	16 (2–261)	9 P, 10 D	NR	10.5 (0)
Mehta et al [63] 2021 (USA)	2000–2020	23 K, 4 L, 2 Pa, 1 H	58.5 (31–76)	20 (NR)	0.988	67.8 (1–188)	7 P, 12 D, 11 other	AMB, 18; azole, 6	29.2 (8.3)
Coccidioidomycosis									
Braddy et al [10] 2006 (USA)	1999–2003	6 K	58 (38–66)^b^	4 (6 W)	2.927	15 (7–24)	4 P, 2 D	Azole, 5	33.3 (33.3)
Vucicevic et al [71] 2011 (USA)	1999–2007	12 L	NR	10 (NR)	3.069	NR	9 P, 3 D	Azole, 12	8.33 (0)
Mendoza et al [32] 2013 (USA)	1999–2011	14 K, 13 L	NR	NR (NR)	1.202	NR	23 P, 4 D	NR	NR
Chaudhary et al [53] 2017 (USA)	1985–2009	11 Lu	46 (13–57)	6 (NR)	5.820	3^b^ (1–64)	10 P, 1 other	NR	63.6 (18.2)
Asbury et al [47] 2019 (USA)	1998–2014	39 K, 4 KP, 1 KL, 1 KLu, 25 L, 7 Lu, 12 H, 1 HL	58 (47–66)	56 (55 W, 3 AA, 24 Hispanic, 7 AI, 1 A 1, other)	1.67	59 (25–100)	37 P, 5 D	AMB, 6; azole, 33	19.8 (1.1)
Histoplasmosis									
Cuellar-Rodriguez et al [54] 2009 (USA)	1997–2007	3 K, 5 H, 3 Lu, 1 L, 1 Pa, 1 KP	45.5 (IQR 38–56.5)	9 (13 W, 1 AA)	0.391	17 (IQR 8.1–46)	NR	AMB, 11> Itra 9; Vori, 2; Itra, 2; Itra > Vori, 1	0 (0)
Assi et al [1] 2013 (USA)	2003–2010	78 K, 24 L, 22 KP, 14 H, 8 Lu, 3 Pa, 1 KLu, 1 SB, 1 KH	48.5 (3–80)	100 (121 W, 23 AA, 3 Latino, 1 As)	NR	27 (1–240)	123 D	AMB, 110; azole, 39	19.08 (9.9)
Luckett et al [100] 2014 (USA)	1999–2012	16 K, 2 H, 2 L, 2 KP, 1 KH	49 (20–67)	8 (17 W, 5 AA)	NR	36 (2–348)	NR	AMB 5; azole, 18	8.70 (8.7)
Guimaraes et al [89] 2016 (Colombia)	1998–2009	10 K	NR	NR	1.101	77 (IQR 8–77)	3 P, 7 D	NR	NR
Mixed cohorts									
Grim et al [57] 2012 (USA)	1996–2008	19 K, 8 L, 3 KP (22 His, 8 B)	54 (19–68)	18 (NR)	0.501	10.5 (1–192)	18 D, 5 other	AMB, 21; azole, 7	13.33 (13.33)
Kauffman et al [184] 2014 (USA)	2001–2006	6 HCT, 33 K, 7 KP, 17 L, 3 Lu, 1 Pa, 3 H (52 His, 9 B, 9 Co)	52 (12–68)^c^	45 (58 W, 8 AA, 4 other)	0.19	11.4 (0–140)	25 P, 45 D	AMB, 43; azole, 24; NA, 3	14.29 (0.109)
Parajuli et al [185] 2018 (USA)	1994–2014	K (14 His, 3 Co, 10 B)	His 43 +/− 17.4	11 (12 W 2 NS)	0.16/100 PY	5.1 +/− 4.2	Majority P	AMB, azole	7.14
			Co 55.6 +/− 25.1	3 (3 W)	0.39/100 PY	4.6 +/− 2.1	3 P	Azole	0
			B 51.7 +/− 12.1	7 (7 W, 3 NS)	0.16/100 PY	1.8 +/− 2.2	8 P	AMB > azole	30
Trinh et al [187] 2018 (USA)	2005–2015	16 K, 6 KP, 3 H, 2 L, 1 Pa (16 His, 9 B, 3 Co)	57 (34–71)	21 (NS)	NR	23 (2–155)	15 D (12 His, 3 B)	AMB, 23; azole, 5	3.33 (NR)

Abbreviations: AA, African American; AI, American Indian/Alaskan Native; As, Asian; AMB, amphotericin B; B, *Blastomyces*; Co, *Coccidioides*; Cut, cutaneous; D, disseminated; H, heart; HCT, hematopoietic transplant; His, histoplasmosis; HL, heart-lung; Itra, itraconazole; K, kidney; KP, kidney-pancreas; KH, Kidney-heart; KL, Kidney-liver; KLu, Kidney-lung; L, liver; Lu, Lung; NR, not reported; NS, not specified; P, pulmonary; Pa, pancreas; PY, person-years; SB, small bowel; Vori, voriconazole; W, white.

^a^No. infections/no. transplants × 100.

^b^Mean.

^c^Includes HCT.

^d^de-escalate.

## DISCUSSION

This review of endemic mycoses in SOT highlights the following: (1) disseminated disease is a frequent syndrome; (2) fever and cytopenias are common manifestations; (3) coinfections with other pathogens may occur; (4) certain endemic fungus have unique features, and (5) relatively high all-cause mortality rates.

The incidence rates of endemic mycoses in SOT recipients appear to be higher than in the general population in endemic regions. For example, population-based studies for blastomycosis [249–251] indicate a prevalence of 0.2–1.94 cases per 100 000 population compared with 1.3–9.8/1000 among SOT recipients in our review [56, 62, 63]. However, this difference needs to be interpreted cautiously, given varying prevalence across regions and underlying populations. Notably, specific risk factors for endemic fungi were reported in only half of cases (131 of 268 [48.9%]). While this could be a reporting issue, it emphasizes the need to elicit a detailed and thorough history from donors and recipients in order to prevent these fungal infections or provide early treatment.

In this review, male SOT recipients were disproportionately affected compared with female recipients (176 of 261 case reports [67.4%]). Although this may be due to behavioral variations (eg, men are generally considered to have more outdoor exposures than women), at least one study [252] that evaluated risk of coccidioidomycosis across different primate species postulates that there may be biological underpinnings of risk, possibly related to hormone production. Another study looked at invasive fungal diseases and reported that some of them were overrepresented in males, including coccidioidomycosis (70%), histoplasmosis (61%) and blastomycosis (66%) [253]. These observations suggest the need for further investigations into the association between biological characteristics (such as sex) and the pathogenesis of invasive fungal diseases, including endemic mycoses.

The majority of endemic mycoses after SOT presented with disseminated syndrome, including a high proportion of fungemia. This is likely due to the underlying immunosuppression. Use of T-cell–depleting agents (34 of 72 [47.2%]) or higher dose immunosuppression (45 of 91 [49.4%]) was documented in many patients. The majority were on MMF which has been previously associated with severe disease (odds ratio, 9.41 [confidence interval, 1.27–66.1]; *P* < .03) [1].

More than two-thirds of SOT recipients developed cytopenias (96 of 135 [71.1%]), probably owing to the disseminated nature of these infections. Cytopenias likely occurred as a result of suppression of normal hematopoiesis by cytokines, direct infection of the bone marrow, or hemophagocytosis [176]. Among the 5 endemic mycoses, histoplasmosis was particularly associated with HLH, a rare syndrome characterized by a hyperstimulated but ineffective natural killer cell immune response with characteristic signs of fever, hepatosplenomegaly, and cytopenias.

Coinfections were reported in some SOT recipients, which could be a phenotypic reflection of the underlying compromised cell-mediated immunity. Conversely, concomitant infections, especially cytomegalovirus, may also depress immune function, further increasing the risk for disseminated fungal infection [254]. The possibility of other infections should be entertained, especially if clinical improvement is slow. In our review, many coinfecting pathogens were intracellular and dependent on a robust T-cell mediated response, which is often impaired among SOT recipients.

This review underscores certain features that are unique to the specific endemic fungi (Table 3). HLH is a unique feature of histoplasmosis. The occurrence of histoplasmosis after SOT is often considered as a de novo infection, while coccidioidomycosis and talaromycosis may result from reactivation of latent infection. Moreover, donor-derived infection appears to be most prominent in coccidioidomycosis. This observation supports the current recommendation to screen all transplant candidates and donors for *Coccidioides* in areas of endemicity and in all exposed individuals identified through a detailed medical history. Recently, guidance regarding screening of seasonal and geographically endemic infections including the endemic fungi for both living and deceased donor screening was published by the Organ Procurement and Transplant Network (OPTN); this document provides relevant information on the identification of potential living and deceased donors who may carry an increased risk of transmitting endemic disease to organ recipients [255].

**Table 3.** Summary of Unique Characteristics of Endemic Mycoses

Characteristic	Blastomycosis	Coccidioidomycosis	Histoplasmosis	Paracoccidioidomycosis	Talaromycosis
Male-female ratio	3.5:1	3:1	2:1	3:1	3:1
Endemic area region	Ohio/Mississippi River Valley	Arizona	Wisconsin, Minnesota, Indiana	South America (Brazil)	Asia, primarily China
Timing after transplant	Variable	Non-DDI, >12 mo; DDI, <1 mo	Usually >12 mo	>12 mo	<12 mo
Fever	In 1/2	In 2/3	In 3/4	In 3/4	In 2/3
Disease	Disseminated > pulmonary > cutaneous	Disseminated >> pulmonary	Disseminated>> pulmonary	Disseminated > pulmonary	Disseminated >> pulmonary
Skin findings	Nontender lesions, can be pruritic	Cutaneous involvement alone rare	Cutaneous involvement alone rare	No reported cutaneous case(s)	No reported cutaneous case(s)
Chest imaging	Infiltrate, nodular, or consolidation	Commonly nodules or infiltrate	May be normal initially; variable	Variable	Variable
Cytopenia	Either pulmonary or disseminated disease	Common with disseminated disease	Common with disseminated disease; HLH reported	Only 1 report	Common with disseminated disease
Fungemia	Not reported	In 1/3	In 1/3	Not reported	In 2/3
Coinfection	Uncommonly reported	Uncommonly reported	May occur	Uncommonly reported	Commonly reported
DDI	None reported	More commonly reported	Reported	None reported	Only 1 so far

Abbreviations: DDI, donor-derived infection; HLH, hemophagocytic lymphohistiocytosis.

Mortality rates differed depending on the endemic mycoses and were highest among those with paracoccidioidomycosis (50%) and coccidioidomycosis (32.3%). The majority of deaths (59 of 71 [83.1%]) occurred among those with disseminated disease, which is likely a reflection of the severity of illness. It is reassuring, however, that among those who survived a disseminated infection, allograft dysfunction or loss was uncommon (9.9%).

Our study has limitations inherent to its study design. First, there may have been duplications across studies (eg, case reports and cohorts), although we attempted to identify and exclude these. Second, the study spanned several decades, and changes in diagnostics, immunosuppressive regimens, and prophylaxis strategies could have affected outcomes. Strategies for antifungal prophylaxis, for example, vary on the type of allograft transplanted and the perceived individual risk, with transplant centers in endemic areas favoring universal prophylaxis (eg, against coccidioidomycosis); this variation in practices could have altered posttransplant clinical course and incidence rates. We also limited our review to publications in English, and important publications in non–English-language journals from endemic regions may have been missed. Third, we excluded reports in which patient-level data could not be extracted, and thus our numbers may underestimate the magnitude of these mycoses. Some relevant data were missing (eg, demographics, outcomes) in some studies, when these data could have added more robust information. Caution should be used when generalizing the results of the study, given that the majority of studies in this review are case reports and case series.

Furthermore, this review is reflective only of data from reported literature, and it does not truly encompass the full picture of endemic mycoses, since there are many more unreported cases in clinical practice. Finally, there were only a few cases of parcoccidioidomycosis and talaromycosis, possibly indicating publication bias. We also did not include the other emerging dimorphic fungi, such as *Emergomyces,* although a recently published systematic review included a few cases in transplant recipients [256]. Despite all these limitations, our study is the only contemporary comprehensive review that organizes the published information on endemic fungi and focuses on the unique features of each endemic fungi among SOT recipients.

In conclusion, our review suggests that endemic mycoses are a threat to SOT recipients with epidemiologic exposures. Severe disseminated disease is a frequent clinical syndrome, and is likely a reflection of the heightened immune suppression in SOT recipients. Cytopenias in a febrile SOT recipient may warrant an evaluation for an underlying endemic fungal infection, particularly in endemic regions. A high index of clinical suspicion for these endemic mycoses is needed to facilitate early diagnosis and treatment, as well as reduce morbidity rates and the relatively high all-cause mortality rates in SOT recipients.

## Supplementary Material

ofae036_Supplementary_Data

## Supplementary Data

Supplementary materials are available at *Open Forum Infectious Diseases* online. Consisting of data provided by the authors to benefit the reader, the posted materials are not copyedited and are the sole responsibility of the authors, so questions or comments should be addressed to the corresponding author.
