# Supplementary material for: Clinical Characteristics and Outcomes of Endemic Mycoses After Solid Organ Transplantation: A Comprehensive Review
Source: Open Forum Infect Dis. 2024 Jan 22;11(3):ofae036. doi: 10.1093/ofid/ofae036 (PMC10913849; doi:10.1093/ofid/ofae036)
Supplement: ofae036_Supplementary_Data [file ofae036_supplementary_data.zip › Supplementary Material, Appendix 1.docx]

Supplementary Material Appendix 1:

A. Search Terms

Database(s): EBM Reviews - Cochrane Central Register of Controlled Trials April 2023, EBM Reviews - Cochrane Database of Systematic Reviews 2005 to May 2023, Embase 1974 to May 2023, Ovid MEDLINE(R) and Epub Ahead of Print, In-Process, In-Data-Review & Other Non-Indexed Citations, Daily and Versions 1946 to May 11, 2023

Search Strategy:

| **#** | **Searches** | **Results** |
| --- | --- | --- |
| 1 | exp Organ Transplantation/ | 715760 |
| 2 | exp Hematopoietic Stem Cell Transplantation/ | 149784 |
| 3 | (haematopoietic or hematopoietic or transplant*).ti,ab. | 1588946 |
| 4 | 1 or 2 or 3 | 1739791 |
| 5 | exp Endemic Diseases/ and exp Mycoses/ | 1132 |
| 6 | (blastomycos* or Coccidioides or "Coccidioides immitis" or "Coccidioides posadasii" or Coccidioidomycosis or coccidiomycos* or "endemic fungi" or "Endemic mycos*" or histoplasmos* or paracoccidiomycos* or Penicilliosis or "Penicillium marneffi" or "T marneffei" or Talaromyces or "Talaromyces marneffei").ti,ab. | 26535 |
| 7 | 5 or 6 | 27093 |
| 8 | 4 and 7 | 1389 |
| 9 | limit 8 to english language [Limit not valid in CDSR; records were retained] | 1342 |
| 10 | (adult or adulthood or adults or centenarian* or elderly or geriatric* or "middle age" or "middle aged" or nonagenarian* or octogenarian* or "old adult*" or "old people" or "old person*" or "older adult*" or "older people" or "older person*" or septuagenarian* or Sextenarian* or "very old").ti,ab,hw,kf. | 20351990 |
| 11 | (newborn* or neonat* or infant* or toddler* or child* or adolescent* or paediatric* or pediatric* or girl or girls or boy or boys or teen or teens or teenager* or preschooler* or "pre-schooler*" or preteen or preteens or "pre-teen" or "pre-teens" or youth or youths).ti,ab,hw,kf. | 10291004 |
| 12 | 11 not 10 | 5994297 |
| 13 | 9 not 12 | 1267 |
| 14 | limit 13 to (letter or conference abstract or editorial or erratum or note or addresses or autobiography or bibliography or biography or blogs or comment or dictionary or directory or interactive tutorial or interview or lectures or legal cases or legislation or news or newspaper article or overall or patient education handout or periodical index or portraits or published erratum or video-audio media or webcasts) [Limit not valid in CCTR,CDSR,Embase,Ovid MEDLINE(R); records were retained] | 370 |
| 15 | 13 not 14 | 897 |
| 16 | remove duplicates from 15 | 542 |

Scopus

1 TITLE-ABS-KEY(haematopoietic or hematopoietic or transplant*)

2 TITLE-ABS-KEY(blastomycos* or Coccidioides or "Coccidioides immitis" or "Coccidioides posadasii" or Coccidioidomycosis or coccidiomycos* or "endemic fungi" or "Endemic mycos*" or histoplasmos* or paracoccidiomycos* or Penicilliosis or "Penicillium marneffi" or "T marneffei" or Talaromyces or "Talaromyces marneffei")

3 LANGUAGE(english)

4 1 and 2 and 3

5 TITLE-ABS-KEY(newborn* or neonat* or infant* or toddler* or child* or adolescent* or paediatric* or pediatric* or girl or girls or boy or boys or teen or teens or teenager* or preschooler* or "pre-schooler*" or preteen or preteens or "pre-teen" or "pre-teens" or youth or youths) AND NOT TITLE-ABS-KEY(adult OR adulthood OR adults OR centenarian* OR elderly OR geriatric* OR "middle age" OR "middle aged" OR nonagenarian* OR octogenarian* OR "old adult*" OR "old people" OR "old person*" OR "older adult*" OR "older people" OR "older person*" OR septuagenarian* OR Sextenarian* OR "very old")

6 4 and not 5

7 DOCTYPE(le) OR DOCTYPE(ab) OR DOCTYPE(ed) OR DOCTYPE(bk) OR DOCTYPE(er) OR DOCTYPE(no) OR DOCTYPE(sh)

8 6 and not 7

9 INDEX(embase) OR INDEX(medline) OR PMID(0* OR 1* OR 2* OR 3* OR 4* OR 5* OR 6* OR 7* OR 8* OR 9*)

10 8 and not 9

Web of Science

1. (haematopoietic or hematopoietic or transplant*) (Topic) and (blastomycos* or Coccidioides or "Coccidioides immitis" or "Coccidioides posadasii" or Coccidioidomycosis or coccidiomycos* or "endemic fungi" or "Endemic mycos*" or histoplasmos* or paracoccidiomycos* or Penicilliosis or "Penicillium marneffi" or "T marneffei" or Talaromyces or "Talaromyces marneffei") (Topic) and English (Language) and Article OR Proceedings Paper OR Review (Document Type)
2. TS=(newborn* or neonat* or infant* or toddler* or child* or adolescent* or paediatric* or pediatric* or girl or girls or boy or boys or teen or teens or teenager* or preschooler* or "pre-schooler*" or preteen or preteens or "pre-teen" or "pre-teens" or youth or youths) NOT TS=(adult OR adulthood OR adults OR centenarian* OR elderly OR geriatric* OR "middle age" OR "middle aged" OR nonagenarian* OR octogenarian* OR "old adult*" OR "old people" OR "old person*" OR "older adult*" OR "older people" OR "older person*" OR septuagenarian* OR Sextenarian* OR "very old")
3. 1 not 2
4. PMID=(0* or 1* or 2* or 3* or 4* or 5* or 6* or 7* or 8* or 9*)
5. 3 not 4
