# Supplementary material for: Clinical Characteristics and Outcomes of Endemic Mycoses After Solid Organ Transplantation: A Comprehensive Review
Source: Open Forum Infect Dis. 2024 Jan 22;11(3):ofae036. doi: 10.1093/ofid/ofae036 (PMC10913849; doi:10.1093/ofid/ofae036)
Supplement: ofae036_Supplementary_Data [file ofae036_supplementary_data.zip › Supplementary Material Appendix 2.docx]

APPENDIX 2: Co-Infections Associated with Endemic Mycoses

| CO-INFECTING PATHOGEN | BLASTOMYCOSIS | HISTOPLASMOSIS | TALAROMYCOSIS |
| --- | --- | --- | --- |
| Bacteria | VRE (2),  MRSA (1), *K. pneumoniae* (1) | *P. aeruginosa* (1), *Capnocytophaga* sp. (1), *S. aureus* (1), *Streptococcus* sp. (1), *C. difficile* (1) | *P. aeruginosa* (2), *A. baumannii* (2), *Enterobacter* sp. (1), *E. coli* (2), *S. aureus* (1) |
| Virus | BK virus (1) | CMV (8), HHV (3), BK virus (1), COVID-19 (1) | CMV (4), HCV (1) |
| Fungi |  | *Candida* sp. (2), PCP (1) | *Candida* sp. (3), *Alternaria* (1), *Aspergillus* sp. (1), PCP (1) |
| Mycobacteria |  | NTM (3)  *M. tuberculosis* (1) | *M. tuberculosis* (1) |

LEGEND: CMV – cytomegalovirus; COVID-19 – Coronavirus-19; HCV – Hepatitis C virus; HHV – human herpes virus; MRSA – Methicillin resistant *Staphylococcus aureus*; NTM – non tuberculous mycobacteria; PCP – pneumocystis jirovecii pneumonia; UTI- Urinary tract infection; VRE – Vancomycin resistant enterococcus
